# The Appointment System Influences Uptake of Cataract Surgical Services in Rwanda

**DOI:** 10.3390/ijerph18020743

**Published:** 2021-01-16

**Authors:** Gatera Fiston Kitema, Priya Morjaria, Wanjiku Mathenge, Jacqueline Ramke

**Affiliations:** 1 Ophthalmology Department, School of Health Sciences, University of Rwanda, Kigali 4285, Rwanda; 2 International Centre for Eye Health, London School of Hygiene & Tropical Medicine, London WC1E 7HT, UK; priya.morjaria@lshtm.ac.uk (P.M.); jacqueline.ramke@lshtm.ac.uk (J.R.); 3 Rwanda International Institute of Ophthalmology (RIIO), Kigali 4285, Rwanda; ciku@email.com; 4 School of Optometry and Vision Science, University of Auckland, Auckland 1010, New Zealand

**Keywords:** cataract, cataract services, health care access, vision impairment, health equity

## Abstract

The aim of this study was to investigate barriers and enablers associated with the uptake of cataract surgery in Rwanda, where financial protection is almost universally available. This was a hospital-based cross-sectional study where potential participants were adults aged >18 years who accepted an appointment for cataract surgery during the study period (May–July 2019). Information was collected from hospital records and a semi-structured questionnaire was used for data collection. Of the 297 people with surgery appointments, 221 (74.4%) were recruited into the study, 126 (57.0%) of whom had attended their appointment. People more likely to attend their surgical appointment were literate, had fewer than 8 children, had poorer visual acuity, had access to a telephone in the family, received a specific date to attend their appointment, received a reminder, and reported no difficulties walking (95% significance level, *p* < 0.05). The most commonly reported barriers were insufficient information about the appointment (*n* = 40/68, 58.8%) and prohibitive indirect costs (*n* = 29/68, 42.6%). This study suggests that clear communication of appointment information and a subsequent reminder, together with additional support for people with limited mobility, are strategies that could improve uptake of cataract surgery in Rwanda.

## 1. Introduction

Globally, in 2015 there was an estimated 36 million blind people and 217 million with moderate or severe vision impairment, 89% of whom lived in low- or middle-income countries [1]. Cataract is the cause of vision loss for one in three blind people globally (12.6 million people) [2], despite cataract surgery being an efficacious intervention [3].

The most recent estimates of vision impairment and blindness in Rwanda are from a national rapid assessment of avoidable blindness (RAAB) of people aged 50 years and above in 2015. This survey estimated the prevalence of blindness (presenting visual acuity worse than 3/60 in the better eye) to be 1.0% (95% CI 0.7–1.4), equating to approximately 18,000 people [4,5]. In addition, the prevalence of moderate and severe vision impairment (presenting visual acuity better than 3/60 and worse than 6/18 in the better eye) was estimated to be 4.4% (95% CI 3.7–5.1), equating to approximately 80,000 people [5].

In Rwanda, few studies have reported barriers to uptake of cataract surgery and there have been no studies on enablers for access to surgery. A study conducted in the Western Province in 2007 reported the main barriers to accessing surgery as lack of awareness (52%), perceived lack of services (16%), and cost of surgery (16%). [6] The two main barriers identified in the 2015 national survey were difficulty in reaching the hospital (33%) and lack of awareness about the services (31%) [5].

In 2019, Rwanda had 16 ophthalmologists for an estimated population of 12 million [7]. Cataract surgery is provided on a regular basis (i.e., at least once weekly) at seven referral hospitals and two private eye hospitals distributed throughout the country. Three of the referral hospitals and the private hospital are in the capital, Kigali. At these hospitals, the waiting list for surgery tends to be short, and often surgery can be booked for the day following diagnosis with operable cataract.

There are a further 37 district hospitals throughout the country that do not have the workforce to provide permanent or weekly cataract surgery but do have at least one permanent ophthalmic clinic officer to provide primary eye care and refractive services. Staff from the referral hospitals deliver regular outreach trips to conduct cataract surgery at each of the district hospitals. In between these surgical visits, the ophthalmic clinic officer generates a list of the people identified with operable cataract who have agreed to have surgery. An appointment is made for the next surgical team visit, which tends to be within the following 6 to 8 weeks.

To address cost as a barrier to health care, Rwanda has a universal health insurance program that includes cataract surgery. Approximately 90% of the population were enrolled in the Community Based Health Insurance in 2016 [8], with participating households paying US$3.50/person/year. Rwanda has classified the population into four socio-economic categories (Ubudehe) based on household income, and financial protection is provided accordingly, with the poorest households (Ubudehe level 1) fully covered by health insurance [9,10]. Cataract surgery is fully subsidized for people in level 1, while people in other levels are required to pay between $3 and $5, which covers the surgery as well as pre- and post-operative care. Despite this financial protection, both ophthalmologists and ophthalmic clinical officers reported ongoing suboptimal uptake of cataract surgical appointments.

The aim of this study was to identify barriers and enablers associated with the uptake of cataract surgery in Rwanda among those who agree to a surgical appointment.

## 2. Materials and Methods

### 2.1. Sample Selection

**Figure 1:**
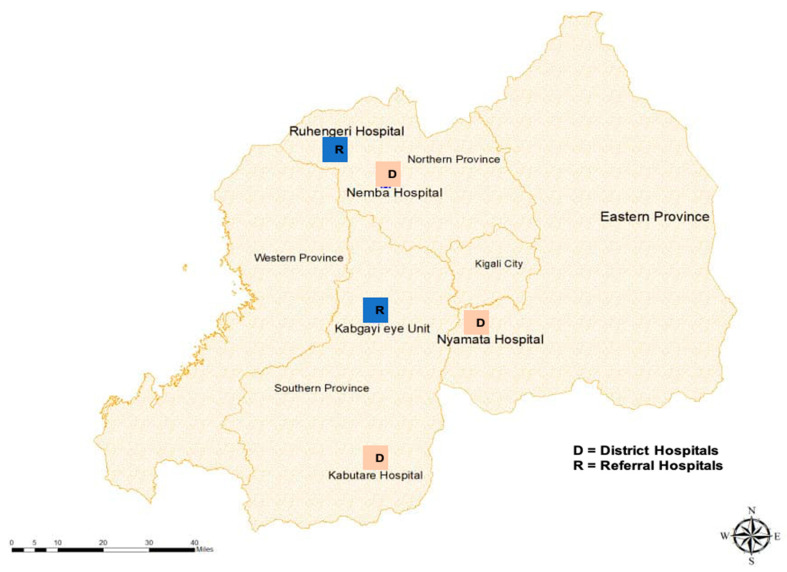
Locations of the two referral (R) and three district (D) hospitals in the study in 2020.

This was a cross-sectional study at five eye care facilities in Rwanda offering cataract surgery between May and July 2019—two referral hospitals and three district hospitals (Figure 1). These hospitals are in three of Rwanda’s five provinces. Potential participants were all adults over 18 years of age with an operable cataract in at least one eye who agreed to undergo surgery and were provided with an appointment for surgery during the study period.

### 2.2. Ethics

Ethical approval for the study was granted by the ethics committee at the London School of Hygiene and Tropical Medicine (Ref: 17256) and the University of Rwanda College of Medicine and Health Sciences (N°: 182/CMHS IRB/2019). Informed consent was obtained from each participant before data collection commenced.

### 2.3. Data Collection

A questionnaire was developed for this study. The outcome variable was attendance at a scheduled cataract surgery appointment (attended/did not attend).

Explanatory variables included:

- Sociodemographic characteristics: Literacy status (literate vs. illiterate), socioeconomic status (Ubudehe Level 1 (poorest) to 4 (wealthiest)), family support status (number of children, escorted to appointment, decision-making), travel time to the eye clinic (hours), cost of transport (US$);
- Whether counselling about surgery was delivered by the ophthalmic clinic officer (counselling received vs. not received);
- Appointment-related factors such as ownership of a mobile telephone in the family, whether a specific date was provided for the surgical appointment, method of receiving appointment information, whether a reminder was provided, the number of days between the diagnosis and surgical appointment;
- General health status (e.g., diabetes, hypertension, HIV and AIDS, rheumatoid arthritis, chronic heart problems);
- Self-reported disability using the Washington group short set of questions on disability [11].

Participants who had not attended their cataract surgery appointment were asked about the main barriers they experienced, and participants who attended their appointment and were severely vision impaired or blind on presentation were asked about what enabled them to attend. Participants could report more than one barrier or enabler.

Questionnaires were translated into Kinyarwanda and a pretest was undertaken on 14 patients at the University of Rwanda eye clinic before the start of data collection. Results from these individuals were not included in the analysis. Data were collected on a tablet and encrypted via Open Data-Kit (https://opendatakit.org/). Demographic information and the visual acuity data were collected from hospital records, surgical registers, and lists of appointments. Attenders were interviewed before they were discharged or during their first postoperative visit; non-attenders were interviewed via telephone.

### 2.4. Analysis

Data were unencrypted and extracted into Microsoft Excel for cleaning and transferred to STATA 15 for analysis. Categorical variables are presented descriptively as counts and percentages and were analyzed to detect sociodemographic and service-related associations with attendance at the surgery appointment.

The Chi-squared test was used to test for trend and logistic regression was performed on variables in relation to the outcomes of interest to test the hypothesis (odds ratios and 95% confidence intervals) for predictors and descriptive statistics in order to present barriers to cataract surgery uptake.

## 3. Results

During the study period, cataract surgery appointments were made for 297 people across the five hospitals, 158 (53.2%) of whom attended their appointment. Across the five hospitals, the attendance rate varied from 41.5% in Nemba to 65.8% in Kabgayi (Table 1).

Of the 297 people with an appointment, 221 (74.4%) were recruited into this study, 126 (57.0%) of whom had attended their appointment (Table 1). Participation in this study was not different between those who attended (126/160; 79%) and did not attend (95/137; 69%) their surgical appointment (*p* = 0.064). One of the reasons for not participating in the study was that some patients did not have time or they could not be reached during the study period. Women comprised 63.6% of people with a surgical appointment, 63.3% of those attending their appointment, and 62.8% of participants in the study.

### 3.1. Uptake of Appointment

Almost 1 in 3 people with an appointment at a referral hospital (44/71; 61.9%) and 1 in 2 people with an appointment at a district hospital (82/150; 54.7%) were unable to attend their appointment; the difference between attendance at the two hospital levels was not statistically significant (0.3).

### 3.2. Factors Associated with Uptake of Appointments

The demographics and service characteristics of those who attended their cataract surgery appointment are provided in Supplementary Table S1. Of the broad range of factors explored, those that were associated with attendance were included in a multivariable logistic regression and they all remained significantly associated with attendance at the surgical appointment (Table 2). People more likely to attend their surgical appointment were literate, had fewer than 8 children, had poorer visual acuity, had access to a telephone in the family, received a specific date to attend their appointment, received a reminder, and reported no difficulties walking (Table 2). Age and sex were not associated with attendance; the 14.0% of participants younger than 60 years had an attendance rate of 71.0% as compared to 54.7% among those 60 years or older, but this difference was not statistically significant (Supplementary Table S1).

### 3.3. Barriers to Uptake of Appointment

The 95 participants who had not attended their surgical appointment during the study period reported a range of barriers. The primary barrier for those with an appointment at a referral hospital (with permanent services) was the cost of the insurance copayment or the indirect cost (*n* = 18/27, 66.7%), followed by lack of an escort (*n* = 6/27, 22.2%). Those with an appointment at an outreach service most commonly reported the barriers to be insufficient information about the appointment (*n* = 40/68, 58.8%) and cost (*n* = 29/68, 42.6%) (Table 3).

Among the 126 participants who attended their appointment, 34 people (27.0%) had vision worse than 6/60 in both eyes (of whom 20 (58.8%) were women). These 34 people—who had experienced vision loss for some time—reported the factors that had enabled their attendance. Receiving adequate appointment information and having family support were the most common enabling factors for participants attending both referral and district hospitals (Table 4).

## 4. Discussion

In this study, we identified that fewer than 3 in every 5 people with operable cataract who accepted an appointment for cataract surgery in Rwanda proceeded to access the appointment. We also identified the sociodemographic and referral-related factors associated with higher uptake of surgery.

The sociodemographic factors were being literate, having fewer than eight children, and having worse presenting visual acuity. The first two factors likely reflect higher socioeconomic status, which is associated with uptake of cataract services [12], while more severe vision loss is also known to motivate service uptake [13]. In contrast to other studies in Sub-Saharan Africa [12], women accessed services at a rate that was not different to men—women were 63.2% of people accessing services in this study, reflecting the higher percentage of women (57.3%) in this age group in the general population [4].

The referral process also involved factors associated with attending surgery—people were more likely to attend if they were given a specific date for their surgical appointment, if they received a reminder about the appointment, if they lived in a household with a telephone (on which to receive a reminder), and if the surgical appointment was in fewer than 10 days following their referral. These findings highlight the importance of communication between service providers and patients with operable cataract and their carers [12]. This importance was reinforced by the extent to which information was reported as a barrier and an enabler to attending the surgery appointment, with insufficient or unclear information the leading barriers reported by people who did not attend their surgical appointment at the district hospitals (Table 4). Mobile telephones—owned by 67% of households in Rwanda in 2016 [14]—are a useful tool for communicating with patients and increasing attendance at appointments. Indeed, telephones enabled increased uptake of referral for cataract surgery in China [15] and for vision screening at a hospital in Kenya [16]. Furthermore, clinical staff could receive context-specific training to deliver counselling that provides clear messages for patients and their carers to promote uptake of interventions for the most socially disadvantaged [17,18].

People were able to attend appointments at the referral hospitals (with permanent ophthalmologists) to a greater extent than the visiting services at the district hospitals. Hospitals with outreach services face the challenge of giving longer appointment lead times and then keeping track of the patients they booked for surgery. Having regular times for surgery and a clear communication channel with the patients could play an important role in increasing the uptake of cataract surgery in Rwanda. Indeed, a study in India reported that regular and reliable outreach or visiting services at the same place to be significant contributors to the uptake of cataract surgical services [19].

People with difficulty walking—which may include the elderly—were less likely to attend their appointment, which is understandable given Rwanda’s mountainous terrain. This finding is in keeping with a recent systematic review that reported that people with disabilities have limited access to health care and increased health expenditure compared to people without disability [20]. Our results reinforce the need for people with disability with an operable cataract be given additional support to attend their surgery appointment. For example, a process could be established whereby eye care counsellors link patients with disabilities to the existing social services fund at the hospital to receive additional financial support to enable attendance at their surgical appointment.

This study focused on a recognized problem in Rwanda’s cataract services, namely identifying reasons why people do not attend their cataract surgery appointment. We have identified specific changes that can be made to the program, and these can be evaluated in a quality improvement cycle. One option is to use the plan–do–study–act cycle [21], which was recently used in India to improve the quality of neonatal care by integrating retinopathy of prematurity services into the government health system [22]. These changes could be incorporated into the work of the technical working group for eye health recently established by the Ministry of Health. This group is coordinated by the Directorate of Planning and includes ophthalmologists and ophthalmic clinical officers from district and referral hospitals. Further research will need to evaluate the measures put in place to increase the uptake of the services and to mitigate the barriers to strengthen what works and readjust what does not work.

Our findings should be interpreted in the context of several limitations. First, the sample frame was drawn from people who had accessed eye care and been identified with operable cataract. This recruitment strategy likely underestimates the extent to which people with operable cataract experience barriers to accessing care, as it excluded those who were unable to access care and be diagnosed with operable cataract. Second, we recruited only 74% of people with a surgical appointment during the study period, and it is possible that the barriers and enablers for the non-participants differed from participants. Indeed, the main reason for being unable to recruit non-attenders was that they could not be contacted—this suggests that the association of owning a mobile phone with attending the appointment may be larger than we identified among participants. Third, data collection relied on self-reporting of the appointment factors, which may involve some degree of recall bias. Further, it is possible other barriers and enablers exist that were not elucidated from our questionnaire. Finally, the interviews with attenders were done at the hospital preceding or following their surgery—information bias is possible if the patients felt they should provide positive responses about their experience.

In 2007, cataract surgical coverage in the Western Province was 47% [6], highlighting that many people who could benefit from surgery had been unable to access care. Since that time, more ophthalmologists and other eye health professionals have been trained, more facilities have been equipped, and regular cataract surgery is now delivered at the district hospital level. This strengthening of the health system contributed to the increase observed in the cataract surgical coverage to 68% in 2015 [5]. Despite these advances, our findings suggest that surgical appointments remain inaccessible for approximately half of Rwandans identified with operable cataract and agreeing to a surgery appointment.

## 5. Conclusions

We have identified modifiable factors associated with increased uptake of cataract surgery—namely clear communication of appointment information and a subsequent reminder, together with additional support for people with limited mobility. These strategies could be further tested and iteratively refined within the national eye care program to ensure cataract surgery is accessible to all Rwandans and that nobody is left behind.

## Figures and Tables

**Table 1:** Summary of the sample frame and participants by attendance at cataract surgery appointment, May–July 2019.

	Sample Frame *n* = 297						Participants *n* = 221					
Facility	Attended Surgical Appointment			Did not Attend Surgical Appointment			Attended Surgical Appointment			Did not Attend Surgical Appointment		
	M	F	T	M	F	T	M	F	T	M	F	T
Referral Hospital												
Kabgayi	19	31	50 (66.6)	10	15	25 (33.3)	12	16	28 (57.1)	8	13	21 (42.9)
Ruhengeri	7	14	21 (60.0)	5	9	14 (40.0)	5	11	16 (72.7)	2	4	6 (27.3)
District Hospital												
Nemba	13	27	40 (42.1)	19	36	55 (57.9)	13	27	40 (50.6)	13	26	39 (49.3)
Nyamata	6	11	16 (55.2)	5	8	13 (44.8)	6	11	17 (68.0)	3	5	8 (32.0)
Kabutare	15	17	32 (51.6)	11	19	30 (48.4)	12	13	25 (54.3)	8	13	21 (45.6)
Total	60	100	160 (53.9)	50	87	137 (46.1)	48	78	126 (57.0)	34	61	95 (43.0)

Note: M = male, f = female, t = total.

**Table 2:** Predictors of uptake of cataract surgery in Rwanda, May–July 2019.

		Total	Attended		OR (95%CI)	*p*-Value *
			*n*	%		
Literate	No	157	80	(51.0)	Ref	
	Yes, with difficulty	24	17	(70.8)	2.3 (0.9–5.9)	0.07
	Yes, easily	40	29	(72.5)	2.5 (1.2–5.4)	0.02
Number of children	8+	63	24	(38.1)	Ref	
	4 to 7	123	79	(64.2)	2.3 (1.2–4.3)	0.008
	<4	35	24	(68.6)	3.1 (1.3–7.4)	0.01
Vision impairment (VI)	Not VI	38	25	(65.8)	Ref	
	Mild	41	22	(58.5)	0.7 (0.1–2.0)	0.5
	Moderate	82	28	(34.2)	0.3 (0.1–0.6)	0.002
	Severe	29	23	(79.3)	2.0 (0.6–6.1)	0.2
	Blind	31	26	(84.0)	2.7 (1.1–10.0)	0.03
Mobile telephone in the family	No	120	45	(37.5)	Ref	
	Yes	101	66	(65.4)	3.6 (2.0–6.3)	<0.0001
Counselling received	Yes	187	100	(54.3)	Ref	
	No	37	26	(70.3)	0.5 (0.2–1.1)	0.08
Specific appointment date provided	No	74	26	(31.4)	Ref	
	Yes	133	100	(75.2)	2.3 (1.3–5.2)	0.004
Appointment reminder provided	No	105	54	(51.5)	Ref	
	Yes	26	23	(88.5)	6.4 (1.8–22.9)	0.004
Number of days between diagnosis and surgical appointment	30+	39	22	(56.4)	Ref	
	10 to 29	38	22	(57.9)	1.4 (0.8–4.1)	0.1
	<10	66	44	(66.6)	1.6 (1.1–4.3)	0.05
Walking difficulties	None	161	99	(61.5)	Ref	
	Some/a lot	60	27	(45.0)	0.5 (0.3–0.9)	0.03

* The *p*-value is for testing the null hypothesis that there is no difference whether a participant attended their cataract surgery appointment or not in relation to the explanatory variable, adjusting for all other variables in the table. N = number, ref = reference.

**Table 3:** Barriers to attending a cataract surgical appointment in Rwanda, May–July 2019 (*n*= 95).

Barriers	Referral Hospital—Permanent Services (*n* = 27)			District Hospital—Outreach Services (*n* = 68)		
	Male	Female	Total (*n*, %)	Male	Female	Total (*n*, %)
Cost	6	12	18 (66.6)	7	22	29 (42.6)
Insufficient/unclear information	2	2	4 (14.8)	13	27	40 (58.8)
Lack of escort	2	4	6 (22.2)	0	7	7 (10.3)
Sickness/other disability	1	2	3 (11.1)	6	4	10 (14.7)
Fear of surgery	2	2	4 (14.8)	1	3	4 (5.9)
Lack of transport	0	0	0 (0.0)	4	5	9 (13.2)
Other	1	0	1 (3.7)	6	7	13 (19.1)

**Table 4:** Enabling factors for people with severe vision impairment or blindness attending their cataract surgical appointment in Rwanda, May–July 2019 (*n* = 34).

Enablers	Referral Hospital—Permanent Services (*n* = 20)			District Hospital—Outreach Services (*n* = 14)		
	Male	Female	Total (*n*, %)	Male	Female	Total (*n*, %)
Received information	4	10	14 (70.0)	5	2	7 (50.0)
Family support	2	9	11 (55.0)	4	4	8 (57.1)
Transport provided	3	1	4 (20.0)	2	2	4 (28.6)
Severity of vision loss	1	4	5 (25.0)	0	2	2 (14.3)
Other	0	0	0 (0.0)	2	3	5 (35.7)

## Data Availability

Data are available upon reasonable request the corresponding author.

## Supplementary Materials

The following are available online at https://www.mdpi.com/1660-4601/18/2/743/s1. Table S1: Description of demographic and service characteristics with attendance at cataract surgery appointment, May–July 2019.
