# Supplementary material for: The Appointment System Influences Uptake of Cataract Surgical Services in Rwanda
Source: Int J Environ Res Public Health. 2021 Jan 16;18(2):743. doi: 10.3390/ijerph18020743 (PMC7830332; doi:10.3390/ijerph18020743)
Supplement: Supplementary file 1 [file ijerph-18-00743-s001.pdf]

International Journal of
Environmental Research and Public Health

MDPI

Table S1. Description of demographic and service characteristics with attendance at cataract surgery appointment, May–July 2019.

Variables		Participants (*n*/%)		Attenders(n/%)		P-value
**Attendance per Hospital**	District Hospital	150	(67.9)	82	(54.7)	0.3
	Referral Hospitals	71	(32.1)	44	(62)	
**Literacy**	Yes, easily	40	(18.1)	29	(72.5)	0.01
	Yes, with difficulties	24	(10.9)	17	(70.8)	
	No	157	(71.0)	80	(51.0)	
**Age (years)**	<60	31	(14.0)	22	(71.0)	0.3
	60-69	66	(29.9)	39	(59.1)	
	70-79	80	(36.2)	42	(52.5)	
	80+	44	(19.9)	23	(52.3)	
**Gender**	Male	81	(36.7)	50	(61.7)	0.28
	Female	140	(63.3)	76	(54.3)	
**Marital status**	Married	152	(68.8)	90	(59.2)	0.49
	Widow /Not married	69	(31.2)	36	(52.2)	
**Location**	Urban	7	(3.2)	4	(57.1)	0.78
	Semi-urban	37	(16.7)	23	(62.2)	
	Rural	177	(80.1)	99	(55.9)	
**Visual acuity (Presenting)**	Blind	31	(14.0)	26	(84.0)	< 0.0001
	SVI	29	(13.1)	23	(79.3)	
	MVI	82	(37.1)	28	(34.2)	
	Mild VI	41	(18.6)	22	(58.5)	
	Normal	38	(17.2)	25	(65.8)	
**SES status (Ubudehe class)**	Very poor	68	(30.8)	38	(55.9)	0.97
	Poor	104	(47.1)	60	(57.7)	
	Middle class and rich	48	(21.7)	28	(57.1)	
**Income status**	Borrow	44	(19.9)	21	(47.7)	0.28
	Use savings	145	(65.6)	83	(57.2)	
	Afford basic needs	32	(14.5)	22	(68.8)	
**Working status**	Professional	7	(3.2)	3	(42.9)	0.4
	Unemployed	7	(3.2)	6	(85.7)	
	Retired	37	(16.7)	21	(56.8)	
	Low skilled	170	(76.9)	96	(56.5)	
**Health insurance status**	No	3	(1.4)	1	(33.3)	0.4
	Yes	218	(98.6)	125	(57.3)	
**Household head status(self)**	No	75	(33.9)	42	(56.0)	0.82
	Yes	146	(66.1)	84	(57.5)	
**Number of children**	<4	35	(15.8)	24	(68.6)	0.009
	4 to 7	123	(55.7)	79	(64.2)	
	8+	63	(28.5)	24	(38.1)	
**Contributors in the decision (Treatment)**	Self	126	(57.5)	69	(54.8)	0.44
	Any form of support	95	(43.4)	57	(60.0)	
**Family support**	No	20	(9.0)	13	(65.0)	0.44
	Yes	201	(91.0)	113	(56.2)	
**Escorted (For appointment)**	No	28	(12.7)	17	(60.7)	0.67
	Yes	193	(87.3)	109	(56.6)	
**Mobile telephone in the family**	No	120	(54.3)	45	(37.5)	<0.0001
	Yes	101	(45.7)	66	(65.4)	
**Specific appointment (days) Time provided**	No	70	(34.5)	26	(31.4)	
	Yes	133	(65.5)	100	(75.2)	0.004
**Counselling received**	No	37	(29.7)	26	(70.3)	0.08
	Yes	184	(53.8)	100	(54.3)	

**Method of receiving Appointment information**	verbal only	68	(59.1)	35	(51.5)	0.2
	Verbal and written	45	(39.1)	24	(53.3)	0.4
	No information provided	2	(1.7)	2	(100.0)	
**Appointment reminder provided**	No	105	(80.2)	54	(51.5)	0.01
	Yes	26	(19.8)	23	(88.5)	
**Time(days) between diagnosis and surgical appointment**	<10	66	(46.2)	44	(66.6)	0.71
	10<29	38	(26.6)	22	(57.9)	
	≥30+	39	(27.3)	22	(56.4)	
**Travel time (Hrs)**	<1	27	(12.2)	15	(55.6)	0.57
	1<2	74	(33.5)	47	(56.8)	
	2<3	80	(36.2)	42	(48.8)	
	>3	39	(17.6)	21	(22.6)	
**Cost of Transport (US$)**	<1	37	(16.7)	26	(70.3)	0.34
	1<2	79	(35.7)	44	(55.7)	
	2-3	80	(36.2)	43	(53.7)	
	>3	25	(11.3)	13	(52.0)	
**Any chronic disease (Infectious/ non-infectious)**	No	71	(51.8)	76	(55.5)	0.55
	Yes	39	(46.4)	50	(59.5)	
**Self-reported disability**	No	71	(51.1)	79	(56.8)	0.94
	Yes	39	(47.6)	47	(57.3)	
**Hearing difficulties**	None	152	(68.8)	86	(56.6)	0.84
	Disability (some + a lot)	69	(31.2)	40	(58.0)	
**Walking difficulties**	None	161	(72.9)	99	(61.5)	0.02
	Disability (some + a lot)	60	(27.1)	27	(45.0)	
